# Direct or Spillover Effect: The Impact of Pure Technical and Scale Efficiencies of Water Use on Water Scarcity in China

**DOI:** 10.3390/ijerph16183401

**Published:** 2019-09-13

**Authors:** Min Li, Kaisheng Long

**Affiliations:** College of Public Administration, Nanjing Agricultural University, 1 Weigang Street, Nanjing 210095, China; 2017109065@njau.edu.cn

**Keywords:** water scarcity, water use pure technical efficiency, water use scale efficiency, panel spatial Durbin model, direct effect, spillover effect

## Abstract

The spatial relationship between water use efficiency and water scarcity has been widely discussed, but little attention has been paid to the impact of the pure technical and scale efficiencies of water use on water scarcity. Using input-oriented data envelopment analysis (DEA) and panel spatial Durbin models (SDM), the direct and spillover effects of different water use efficiencies on water scarcity from 2007 to 2016 in China were examined at the regional scale. The results show that the water use pure technical efficiency had significantly negative direct effects on water scarcity; however, the water use scale efficiency did not have a similar effect. The improvement in water use pure technical efficiency in one region could aggravate the water scarcity in neighboring regions through spatial spillover effects, but the same effect was not observed between the water use scale efficiency and water scarcity. Finally, we propose solutions to improve the water use efficiency to reduce the water scarcity.

## 1. Introduction

Scarcity and efficiency are two fundamental themes of economics [1]. It is generally accepted that a scarce resource should be explored based on its efficiency. Water resources are one of the scarcest resources in the world, therefore, improving water use efficiency is a crucial aspect in addressing water scarcity [2,3,4,5]. However, in most cases, increases in water use efficiency are rarely associated with increased water availability on larger scales [6]. Water consumption increases with growth in the production of goods and services [7]. Though water use efficiency has improved, the spatial and seasonal mismatch of water resources is not yet been resolved [8]. Research has found that no significant causal mechanism exists between water use efficiency and water scarcity from a spatial perspective [9]. Thus, more attention should be paid to explore the complex efficiency–scarcity nexus of water resources.

Water use efficiency can be viewed as a kind of overall technical efficiency [10], which can be decomposed into pure technical and scale efficiencies [11]. The pure technical efficiency reflects the management ability to organize the inputs in the production process, whereas the scale efficiency reflects the management ability to choose the optimum size of resources, i.e., choosing a proper production scale to attain the expected output [12]. Some studies proved that overall loss of resource efficiency use might be due to a deficiency in pure technical or scale efficiency [13,14]. Hence, resolving water use efficiency into pure technical and scale efficiencies provides a proper method to optimize water resource allocation. Resources use efficiency can sometimes affect resources allocations in neighboring regions through spillover effects [15,16]. Therefore, the direct and spatial spillover effects should be considered as aspects of the spatial relationship between water use efficiency and water scarcity.

As the depletion of water resources is growing in most developing countries, increasing pressure on their water resources [17], improving water use efficiency in developing countries with scarce water resources is important [18]. China’s water resources are characterized by low per capita availability and extremely uneven spatial distribution [19,20]. In general, agriculture and industrial sectors and households are the main water consumers [21]. With the growing economy, and increasing urbanization and living standards, the conflict between water supply and demand is becoming increasingly serious, leading to a prevalent water crisis in China [21,22]. The Chinese government has taken measures to reduce water scarcity, such as introducing innovative technology and optimizing legislation systems [23,24]. Although everything possible has been implemented to address the water situation in China, water supply system still faces challenges [21]. Water consumption is still rising due to increases in water demand for production and consumption, as well as in net virtual water exports [25]. Currently, water resource has become one of the constraining factors hindering China’s sustainable development, and water security is expected to worsen until 2030 [20]. With the aim of reducing water scarcity through improving water use efficiency, we attempted to examine the relationship between water use technical and scale efficiencies and water scarcity from a spatial perspective in China.

To better understand the direct and spillover impacts of water use technical and scale efficiencies on water scarcity, we addressed the following questions: Does water use technical efficiency or scale efficiency affect water scarcity? Do any differences exist between their impacts? We then conducted several spatial analyses and report the work as follows: Section 2 presents the water scarcity index, water use efficiency index, and other control variables. We describe the spatial econometrics models used herein. Section 3 presents the results of the panel spatial Durbin model and the direct and spillover effects of technical and scale efficiency on water scarcity. Section 4 provides a conclusion and recommendations for reducing water scarcity.

## 2. Materials and Methods

### 2.1. Water Scarcity Index (WSI)

Water is a renewable [26] as well as an irreplaceable resource [27]. The availability of water resources varies in time and space [26]. As water consumption in different industries is increasing rapidly, water consumption will exceed the availability of water resources, and therefore water scarcity is inevitable. The Falkenmark water stress indicator has been widely used to determine how much water is available and how we can best benefit from the available water [28]. Others measured water scarcity by comparing annual renewable water supply and annual demand [29,30,31]. Given the complex factors influencing water resources, some researchers have established a global water scarcity risk assessment framework, for example the incorporating hydro-climatic variability and socioeconomic growth [32,33]. To achieve sustainable environment development, indices incorporating environmental water requirements have also been introduced [34].

Considering the availability and reliability of data, the use-to-resource ratio, which is the ratio of annual water withdrawals to annual renewable water resources [31], was adopted in this study. Use-to-resource ratio implies that water scarcity is strongly affected by water consumption and availability. Raskin et al. [31] defined water scarcity by four classes ranging from a value of 0 for no stress to 3 for absolute scarcity based on their use-to-resource ratio study (Table 1).

### 2.2. Water Use Efficiency Index (WUEI)

Water use efficiency often refers to the ratio between water input and useful economic/product output [35], and is measured by either a parametric method, such as stochastic frontier analysis (SFA), or by a non-parametric measure, such as data envelopment analysis (DEA). DEA can be used to evaluate the efficiency of multiple inputs and outputs. DEA has been widely used in different areas, such as energy and health efficiency [36] and environmental–economic efficiency [37]. The major advantage of DEA is that it does not assume any a priority function relationship between the inputs and outputs [38]. The DEA model under the constant returns-to-scale (CRS) assumption was proposed by Charnes et al. [39]. As is shown in previous studies [40], overall water use technical efficiency (TE) can be measured using this model. However, the efficiency calculated in the CRS model includes technical and scale effectiveness. We cannot distinguish pure technical and scale efficiencies when water use is inefficient. Later, Banker et al. [41] introduced a DEA model under the variable returns-to-scale (VRS) assumption. This model specializes in calculating pure technical efficiency (PTE) by considering the decision making units (DMUs) as the units on the efficient production frontier with the same size. Efficiency can be calculated using CRS or VRS models based on input- or output-oriented perspectives, which aim to minimize inputs or maximize outputs, respectively. With the goal of reducing water scarcity by decreasing water withdrawals, we adopted input-oriented CRS and VRS models in this study to evaluate overall water use efficiency, pure technical efficiency, and scale efficiency.

The CRS model is presented here for a case with available data on *i* (*i* = 1, 2, …, *m*) inputs and *r* (*r* = 1, 2, …, *s*) outputs for each of the *j* (*j* = 1, 2, …, *n*) regions. For *j* regions, the inputs and outputs are re-presented by the column vectors *X_j_* (*X_j_* = *x*_1*j*_, *x*_1*j*_, …, *x_mj_*) and *Y_j_* (*Y_j_* = *y*_1*j*_, *y*_1*j*_, …, *y_rj_*) respectively. The overall technical efficiency (*TE*)—the overall water use efficiency— is calculated using Equation (1):

(1) $$\left(D^{I}{}_{CRS}\right)\{\begin{array}{c} nin\theta ,\\ \overset{n}{\underset{j=1}{\sum }}X_{j}\lambda_{j}\leq {\theta X}_{0j},\\ \overset{n}{\underset{j=1}{\sum }}Y_{j}\lambda_{j}\geq Y_{0j},\\ \lambda_{j}\geq 0 \end{array}$$

where θ represents the efficiency score of each of the *j* regions and θ∈[0, 1], and $\lambda_{j}$ is the vector of input and output weights. To calculate water use pure technical efficiency, we imposed a restriction $\sum_{j=1}^{n}\lambda_{j}=1$ on Equation. (1) to establish a DEA model under the VRS assumption. The water use pure technical efficiency (*PTE*) can be measured using Equation (2):

(2) $$\left(D^{I}{}_{VRS}\right)\{\begin{array}{c} min\theta ,\\ \sum_{j=1}^{n}X_{j}\lambda_{j}\leq {\theta X}_{0j},\\ \sum_{j=1}^{n}Y_{j}\lambda_{j}\geq Y_{0j},\\ \sum_{j=1}^{n}\lambda_{j}=1,\\ \lambda_{j}\geq 0 \end{array}.$$

According to Banker et al. [41], scale efficiency results from the differences in the production frontiers based on CRS and VRS assumptions. This means that water scale efficiency can be calculated based on overall water use and pure technical efficiency. Therefore, water use scale efficiency (*SE*) can be computed in the following equation:

*SE* = *TE*/*PTE* (3)

where *SE* is the water use scale efficiency, which reflects the most efficient scale of input for attaining the expected production.

According to the above models of the water use efficiency index (WUEI), we had to select proper input and output indices. With respect to output, economic output is a commonly employed index [35] and we chose gross domestic production (GDP) as its substitution. Economic development depends, to a large extent, on resources, labor, and capital inputs. Therefore, we considered population, fixed investment, and total water use of different industries as inputs and used them to assess the WUEI (Table 2).

### 2.3. Model Specification

Previous studies have found that water availability and demand have the inherent nature of high spatial and temporal variability [42]. Water use efficiency also has significant spatial spillover effects [43] and changes across a range of both the temporal and spatial scales [44]. Hence, water scarcity is not only affected by the local water use efficiency, but also by its neighboring regions. Some spatial models are typical; the spatial lag model (SLM), spatial error model (SEM), and spatial Durbin model (SDM) can be used to explore the relationship between water scarcity and water use efficiency [45]. Here, we employed the panel spatial Durbin model (SDM) to estimate spatial effects because even if the true data generation process contains spatial lag or spatial error, the SDM can still produce unbiased coefficient estimates [46]. The extension of the SDM to panel data allows the exploitation of the usual advantages of panel data [47], which improves the efficiency of estimation [48]. Because only a 10-year period was investigated in this study, we did not consider time. Therefore, the SDM takes the form:

(4) $${WSI}_{nt}=\rho W\times {WSI}_{nt}+\beta \times {WUEI}_{nt}+\theta W\times {WUEI}_{nt}+\mu_{n}+\varepsilon_{nt}$$

where *n* denotes the region; *t* represents the year; *W* denotes the non-negative spatial weight matrix; *W* × *WSI* and *W* × *WUEI* are the spatially lagged dependent and independent variables, respectively; ρ denotes the spatial autocorrelation coefficient; and β and θ are spatial regressive coefficients. $\mu_{n}$ and $\varepsilon_{nt}$ are individual fixed effects and error term, respectively. To improve the efficiency of the estimation, we introduced some control variables into the model. The other variables also impact on water scarcity. Therefore, the specific econometric model can then be constructed as:

(5) $${WSI}_{nt}=\rho W\times {WSI}_{nt}+\beta \times {WUEI}_{nt}+\theta W\times {WUEI}_{nt}+\alpha_{1}\times C_{nt,k}+\alpha_{2}W\times C_{nt,k}+\mu_{n}+\varepsilon_{nt}$$

where *C* (*C* = 1, 2, …, *k*) represents the control variables. 

Then, choosing the proper spatial weight matrix in the SDM is critical. The spatial weight matrix *W* has various forms, and it is specifically set according to different research aims. Due to the existence of both common boundaries and common corners between neighboring regions, *W* was created by GeoDa 1.6.7 based on the queen criterion as described by Anselin [49]. Therefore, *W* is a binary spatial weighting matrix and commonly row-standardized:

(6) $$W=\{\begin{array}{c} 1,\;\mathrm{when}\;\mathrm{two}\;\mathrm{regions}\;\mathrm{are}\;\mathrm{adjacent}\\ 0,\;\mathrm{when}\;\mathrm{two}\;\mathrm{regions}\;\mathrm{are}\;\mathrm{not}\;\mathrm{adjacent} \end{array}$$

However, the panel SDM results cannot be interpreted as partial derivatives in the typical regression model. As suggested by LeSage and Pace [50], we referred to the summary measures of direct and spillover effects to assess the magnitudes of impacts arising from changes in water use pure technical and scale efficiencies, as well as control variables. Direct effects, including the spatial feedback effect, refer to the impact of explanatory variables on *WSI*. Spillover effects, also called indirect effects, denote that the explanatory variables affect the *WSI* in surrounding regions. The specific derivation processes are shown:

(7) $${WSI}_{nt}={\left(I_{n}-\rho W\right)}^{-1}(\beta \times {WUEI}_{nt}+\theta W\times {WUEI}_{nt}+\alpha_{1}\times C_{nt,k}+\alpha_{2}W\times C_{nt,k}+\mu_{n}+\varepsilon_{nt})$$

(8) $$\left[\frac{\partial {E(WSI}_{t})}{\partial {WUEI}_{t}},\ldots ,\frac{\partial {E(WSI}_{t})}{\partial {WUEI}_{t}}\right]=\left[\begin{array}{ccc} \frac{\partial {E(WSI}_{1t})}{\partial {WUEI}_{1t}} & \cdots & \frac{\partial {E(WSI}_{1t})}{\partial {WUEI}_{nt}}\\ \vdots & \ddots & \vdots \\ \frac{\partial {E(WSI}_{nt})}{\partial {WUEI}_{1t}} & \cdots & \frac{\partial {E(WSI}_{nt})}{\partial {WUEI}_{nt}} \end{array}\right]={\left(I_{n}-\rho W\right)}^{-1}({\beta I}_{n}+\theta W)$$

(9) $$\left[\frac{\partial E(WSI)}{\partial C_{t,k}},\ldots ,\frac{\partial E(WSI)}{\partial C_{t,k}}\right]=\left[\begin{array}{ccc} \frac{\partial {E(WSI}_{1t})}{\partial C_{1t,k}} & \cdots & \frac{\partial {E(WSI}_{1t})}{\partial C_{nt,k}}\\ \vdots & \ddots & \vdots \\ \frac{\partial {E(WSI}_{nt})}{\partial C_{1t,k}} & \cdots & \frac{\partial {E(WSI}_{nt})}{\partial C_{nt,k}} \end{array}\right]{=(I_{n}-\rho W)}^{-1}(\alpha_{1}I_{n}+\alpha_{2}W)$$

The direct and spillover effects of water use efficiency and control variables can be calculated by Equations (8) and (9), respectively. These equations show that the main diagonal elements represent the direct effects (the own-partial derivatives), and the mean sum row of the non-diagonal elements represents the spillover effects (the cross-partial derivatives). Due to the differences in regions and the independence of time, we used average effects in this study. Average direct effects can be calculated from the average of the elements on all diagonals of the partial derivative matrix. The average spillover effects can be measured from the average of all non-diagonal elements of the partial derivatives matrix.

### 2.4. Control Variables

Water scarcity is not only affected by water use efficiency, but also by other factors such as socioeconomic conditions and water supply and demand. We introduce a set of control variables to improve the effectiveness of estimations. Increasing water use is amplified by urbanization and economic growth [51], and water stress is expected to intensify with rapid population and rapid economic growth [52]. Therefore, the per capita gross domestic production (*lnPGDP*) exerts a strong influence on water use intensity. R&D represents another important factor affecting water scarcity [53]. We used research and development expenditure (*lnRD*) to reflect R&D. As discussed by Falkenmark et al. [28], the water available in a country or region is derived from the water cycle, which considerably affects total water resources. Water management problems are directly related to the population flow, and an important nexus exists between population and water resources [54]. Therefore, per capita water resource (*lnPERWATER*) has a significant impact on water scarcity. Precipitation (*lnPRE*) is the main source of available fresh water resources for most regions. Scarcity of water resources results from the start of a precipitation deficit, and successive components of the hydrological cycle are affected [55]. We finally selected these two factors as control variables. Water scarcity is determined by water consumption. Agriculture, mostly irrigated agriculture, is the largest consumer of water [56]. Globally irrigated agriculture accounts for 70% of the global water demand [57]. Thus we take the ratio of agricultural water consumption to total available water resources (*lnAGRWATER*) as a control variable.

### 2.5. Data Sources and Description

Data used to calculate water use efficiency, water scarcity, and control variables were collected from the *China Statistical Yearbook*, the *China Statistical Yearbook on Science and Technology* and the *China Statistical Yearbook on Environment* [58,59,60]. Due to data availability, we chose to use the data of 31 regions in mainland China (including four municipalities, five autonomous regions, and 22 provinces) from 2007 to 2016. Specifically, GDP, fixed investment, population, and per capita GDP data were collected from the *China Statistical Yearbook* (2008–2017) [58]. The inflation effect has not been taken into account in this study due to the short time series, GDP, fixed investment and per capita GDP were thus expressed in nominal yuans. R&D data were collected from the *China Statistical Yearbook on Science and Technology* (2008–2017) [59]. The other indices were all collected from the *China Statistical Yearbook* *on Environment* (2008–2017) [60]. Table 3 lists the descriptive statistics of all the variables used in this study. All control variables are in logarithmic form to eliminate heteroscedasticity and to stabilize the panel data.

## 3. Results and Discussion

We estimated the impacts of water use pure technical and scale efficiencies on water scarcity through SDM using Stata 15.0 (StataCrop LLC, 4905 Lakeway Drive, College Station, TX 77845, USA). Table 4 shows the results of the ordinary least-squares (OLS) and SDM estimates with fixed-effects and random-effects.

From Table 4, since *rho* is significantly different from zero in most cases, the OLS estimates are biased and inconsistent. Theoretically, the regions selected in all of models are regarded as a fixed sampling, so the fixed effect model is more suitable. The results of the Hausman specification test shown in Table 4 also support the fixed-effects specification.

As discussed above, to assess the impacts arising from changes in water use pure technical efficiency, scale efficiency, and control variables, we measured the direct and spillover impacts based on Equations (7) and (8). The results are presented in Table 5 and Table 6. In particular, we measured the effects of overall technical efficiency of water use on water scarcity. Table 5 and Table 6 show that both the direct and spillover effects of overall water use technical efficiency are not significant, indicating that no causal relation or spatial spillover effects exist between water use efficiency and water scarcity. Therefore, an overall efficiency-oriented policy may not be sufficient for optimizing water use [40]; we need to further explore the reason for water use inefficiency caused by pure technical or scale efficiency.

### 3.1. Direct Effects of Water Use Efficiency on Water Scarcity

Table 5 demonstrates that the coefficient of water use pure technical efficiency (*PTE*) is negative at the 10% significance level, suggesting that water use pure technical efficiency has a negative impact on water scarcity. Generally, an increase in water use pure technical efficiency means that the input structure is more efficient. According to the results of water use pure technical efficiency based on the DEA model, input slacks are mainly due to water consumption. Therefore, the improvement in pure technical efficiency indicates that the increases in capital, labor, and technology are replacing water consumption. A decrease of water consumption means that more water resources will be available. The more the available water resources increase, the greater the water resource supplies. From this result, we think that water scarcity can be effectively reduced by the increase in water use technical efficiency.

Compared with the direct effect of water use pure technical efficiency on water scarcity, the effect of water use scale efficiency (*SE*) is not significant. The increase in water use scale efficiency indicates that the actual production scale is closer to the optimal production scale and that no water input is redundant. Preventing people from consuming water and related services is usually difficult, as water resources are usually considered public goods [61]. Thus, water resources saved in one industry are consumed by other industries. According to the results of water use scale efficiency calculated by the DEA model, water scale efficiency is high in general, regardless of year. We observed that many differences between the actual production scale and the optimal production scale. Therefore, the increase in water use scale efficiency would not result in significant changes in water demand.

### 3.2. Spillover Effects of Water Use Efficiency on Water Scarcity

Table 6 shows that the spillover effect of water use pure technical efficiency (*PTE*) is positive at the 5% significance level. This means that water use pure technical efficiency could aggravate water scarcity in neighboring regions. From the perspective of the “new” new economic geography (NNEG) theoretical framework [62], technology agglomeration can lead to a core–periphery (C–P) distribution of resources. The improvement in water use pure technical efficiency indicates that more advanced technologies are employed in production processes, helping to establish a technology agglomeration area. Therefore, technology-intensive water use in one region will lead to high water consumption industries staying and agglomerating in the neighboring regions. As a result, although water use pure technical efficiency may improve in one region, the excessive water use of its neighboring regions will not be reduced, and water scarcity will increase to some extent.

However, the spillover effect of water use scale efficiency (*SE*) is not significant, as shown in Table 6. This means that water scarcity in one region cannot be affected by the water use scale efficiency of its neighboring regions. We provide two reasons for this result. First, no significant water use scale efficiency discrimination exists between regions, as calculated by the DEA model. Thus, the change in water use scale efficiency caused by neighboring regions is limited, which cannot strongly influence water consumption either. Second, the adjustment of the production scale only affects the same industries in neighboring regions rather than the whole society. Therefore, a change in water consumption denotes only part of water consumption rather than overall water resource inputs. As a result, water scarcity cannot be significantly affected by the spillover effect of water use scale efficiency.

### 3.3. Effects of Control Variables on Water Scarcity

With respect to socioeconomic and technological factors, we found that the direct effects of research and development expenditure (*lnRD*) are significantly negative, whereas those of per capita gross domestic production (*lnPGDP*) are significantly positive. The investment into R&D is an important method of improving water use pure technical efficiency. An increase in R&D means that more advanced technologies (rather than more water resources) are invested into production. However, the increase in GDP denotes the enlargement of production. According to the change in the RTS, increased production requires more water resources in China to a large extent.

For available water resource factors, the direct effects of per capita water resources (*lnPERWATER*) are significantly negative, whereas the spillover effects of precipitation (*lnPRE*) are significantly negative. The increases in per capita water resources and precipitation indicate the improvement in water resource availability, and water scarcity will decrease correspondingly as long as water usage remains stable. The results also mean that the water scarcity of one region is determined by the local climate and geographical conditions as well as those of the neighboring, as discussed by Ahmed et al. [63]. A possible reason for this result is that an increase in precipitation in one region will be transferred to other regions by surface and underground runoff, indirectly changing water allocation in the neighboring regions. In summary, we infer that, as water saving technologies are not widely adopted in China, not only water use efficiency but also other factors affecting water demand and supply are major determinants of water scarcity.

### 3.4. Comparison with Previous Lliteratures

The results obtained in this study indicate that water use pure technical efficiency and scale efficiency have different effects on water scarcity. Compared with previous research conducted by Long and Pijanowski [9], who found no significant causal mechanisms between water use efficiency and water scarcity, and by Sun et al. [42], who found significant spatial spillover effects of water resource use efficiency in China, this study provides a useful insight for understanding the relationship between different aspects of water use efficiency and water scarcity. Water use pure technical efficiency and scale efficiency refer to different water resource allocation mechanisms, so their effects on water scarcity should be examined separately. Our results promote the development of the efficiency–scarcity nexus of water resources from a multi-efficiency and multi-effect perspective.

This study only contains one decade’s worth of data from 2007 to 2016; we thus realize that our results are part of the efficiency–scarcity nexus of water resources and are not representative of a long-standing cognition. In addition, we only discussed the impact of water use pure technical efficiency and scale efficiency on water scarcity, so the results do not reflect the degree of water use efficiency affected by scarcity and cannot be seen as an improvement on existing findings (see Varghese et al. [64]). We think that other results regarding the efficiency–scarcity nexus of water resources could be obtained as the time and study areas change.

## 4. Conclusions and Recommendations

In this study, we divided overall water use efficiency into water use pure technical and scale efficiencies and distinguished their impacts on water scarcity. Our results exceed those previous studies regarding the efficiency–scarcity nexus of water resources from a spatial perspective. Specifically, water use pure technical efficiency has significant negative direct effects on water scarcity; however, water use scale efficiency was not found to have a similar effect. This study also shows that water use pure technical efficiency has significant positive spillover effects, indicating that water use pure technical efficiency could aggravate water scarcity in neighboring regions. No spillover effect was found between water use scale efficiency and water scarcity. Besides water use pure technical efficiency, other factors including economy, technology, and precipitation are crucial determinants of water scarcity. These results are not only specific to the study area, but also should be paid attention to when examining efficiency–scarcity nexus of water resources elsewhere.

These findings imply that we should pay more attention to the effects of water use efficiency on water resource allocation as well as consider other factors affecting water demand and supply. This would help us to break the paradox of efficiency and maintain the sustainability of water resources. We present the following recommendations that we think could reduce water resource consumption and address water scarcity by improving water use efficiency. Firstly, promoting technology innovation in the whole country, not only in some regions, is necessary. Due to the positive spillover effect of water use pure technical efficiency on water scarcity, water-saving technology innovation in one region will not be beneficial to the sustainability of water resources of other regions. We therefore must promote water-saving technology innovation at the national scale, not at the local scale. Secondly, as water use scale efficiency has not helped reduce water scarcity, water saving policies regarding decreased water requirements and consumption should be formulated and implemented. In particular, a suitable water price, punishment for water-wasting behavior, and a perfect legislation system of water resource conservation would be indispensable. Finally, as economic and technical factors are influencing factors of water scarcity, we think that high-quality economic development and increasing R&D inputs are urgently required to reduce water scarcity in transitional China.

## Figures and Tables

**Table 1:** Water scarcity index (*WSI*) definition.

Classification	No Stress	Stress	Scarcity	Absolute Scarcity
Use-to-resource ratio	<0.1	0.1–0.2	0.2–0.4	>0.4
Value	0	1	2	3

**Table 2:** Description of the indices selected to assess the water use efficiency index (WUEI). GDP denotes gross domestic product.

	Indices	Units
Inputs	*i*_1_: population	10^4^ person
	*i*_2_: fixed investment	10^8^ yuan
	*i*_3_: agricultural water use	10^8^ m^3^
	*i*_4_: industrial water use	10^8^ m^3^
	*i*_5_: living water use	10^8^ m^3^
	*i*_6_: ecological water use	10^8^ m^3^
Output	*r*: GDP	10^8^ yuan

**Table 3:** Descriptive statistics of variables used.

Variables	Mean	SD	Min	Max	No. Observations
*Water scarcity*	1.948	1.105	0.000	3.000	310
*PTE*	0.788	0.195	0.414	1.000	310
*SE*	0.936	0.103	0.455	1.000	310
*TE*	0.734	0.195	0.399	1.000	310
*lnPGDP*	10.448	0.553	8.841	11.680	310
*lnRD*	14.066	1.574	8.849	16.829	310
*lnPERWATER*	7.197	1.497	4.288	11.981	310
*lnPRE*	7.154	1.164	4.009	8.912	310
*lnAGRWATER*	4.054	0.395	2.583	4.556	310

Notes: Water scarcity denotes the use-to-resource ratio. *PTE* denotes water use pure technical efficiency. *SE* denotes water use scale efficiency. *TE* denotes overall water use technical efficiency. *PGDP* is per capita gross domestic production. *RD* is research and development expenditure. *PERWATER* is per capita water resources. *PRE* is precipitation. *AGRWATER* is the ratio of agricultural water consumption to total available water resources.

**Table 4:** Results of the ordinary least-squares (OLS) and spatial Durbin model (SDM) estimates with fixed-effects (FE) and random-effects (RE).

Model	OLS	SDM-FE	SDM-RE	OLS	SDM-FE	SDM-RE
Water use efficiency	PTE	PTE	PTE	SE	SE	SE
*Rho*		0.136 *	0.226 ***		0.115	0.220 ***
*PTE*	−0.459 *	−0.507 **	−0.466 *			
*SE*				0.307	0.407 *	0.464 *
*lnPGDP*	0.240	0.405 *	0.265	0.251	0.418 *	0.141
*lnRD*	−0.265 **	−0.246 *	−0.209 **	−0.256 *	−0.281 **	−0.198 **
*lnPERWATER*	−0.738 ***	−0.835 ***	−0.861 ***	−0.731 ***	−0.796 ***	−0.847 ***
*lnPRE*	−0.375 *	−0.118	0.225	−0.384 *	−0.147	0.200
*lnAGRWATER*	0.105	0.187	0.220	0.0602	0.171	0.169
Cons_	11.11 ***		5.663 ***	10.39 ***		6.200 ***
*W.PTE*		1.278 **	1.175 **			
*W.SE*					−0.739	−0.840 *
*W.lnPGDP*		−0.0337	0.0378		−0.0372	0.065
*W.lnRD*		−0.0341	−0.0453		−0.0324	−0.00184
*W.lnPERWATER*		0.432 *	0.300		0.410 *	0.325
*W.lnPRE*		−0.792 *	−0.400		−0.788 *	−0.436
*W.lnAGRWATER*		−0.712	0.0535		−0.690	0.290
sigma2_e		0.0772 ***	0.0881 ***		0.0784 ***	0.0898 ***
R^2^	0.5312			0.5221		
Hausman		13.45 **			23.07 ***	

Notes: *, **, and *** denote statistical significance at the 10%, 5%, and 1% levels, respectively. *W.* variables denote spatially lagged variables. *rho*, Cons_, sigma2_e and *R^2^* denote spatial autoregressive parameters, constants, standard deviation of idiosyncratic errors and the coefficient of determination, respectively. Hausman denotes the results of the Hausman specification test.

**Table 5:** Direct effects of water use efficiency on water scarcity.

Explanatory Variables	Model-*PTE*	Model-*SE*	Model-*TE*
*PTE*	−0.460 *		
*SE*		0.399	
*TE*			−0.0534
*lnPGDP*	0.395 *	0.409 *	0.392 *
*lnRD*	−0.234 *	−0.269 **	−0.256 **
*ln* *PERWATER*	−0.826 ***	−0.788 ***	−0.812 ***
*lnPRE*	−0.141	−0.167	−0.126
*lnAGRWATER*	0.182	0.169	0.217

Notes: *, **, and *** denote statistical significance at the 10%, 5%, and 1% levels, respectively.

**Table 6:** Spillover effects of water use efficiency on water scarcity.

Explanatory Variables	Model-*PTE*	Model-*SE*	Model-*TE*
*PTE*	1.334 **		
*SE*		−0.739	
*TE*			0.299
*lnPGDP*	0.00573	−0.00708	0.0569
*lnRD*	−0.0622	−0.0582	−0.0986
*lnPERWATER*	0.364	0.357	0.368
*lnPRE*	−0.909 **	−0.886 *	−0.926 **
*lnAGRWATER*	−0.705	−0.674	−0.808

Notes: *, **, and *** denote statistical significance at the 10%, 5%, and 1% levels, respectively.
